# Multidisciplinary teams, and parents, negotiating common ground in shared-care of children with long-term conditions: A mixed methods study

**DOI:** 10.1186/1472-6963-13-264

**Published:** 2013-07-08

**Authors:** Veronica M Swallow, Ruth Nightingale, Julian Williams, Heather Lambert, Nicholas JA Webb, Trish Smith, Lucy Wirz, Leila Qizalbash, Laura Crowther, Davina Allen

**Affiliations:** 1 School of Nursing, Midwifery and Social Work, Faculty of Medical and Human Sciences, Manchester Academic Health Sciences Centre, University of Manchester, Oxford Road, Manchester, M13 9PT, UK; 2 Royal Manchester Children’s Hospital, Central Manchester University Hospitals NHS Foundation Trust, Oxford Road, Manchester, M13 9WL, UK; 3 The Great North Children’s Hospital, Newcastle upon Tyne Hospitals NHS Foundation Trust, Queen Victoria Road, Newcastle upon Tyne, NE1 4LP, UK; 4 Cardiff School of Nursing and Midwifery Studies, Cardiff University, Newport Road, Cardiff, CF24 0AB, UK; 5 School of Education, University of Manchester, Oxford Road, Manchester, M13 9PL, UK; 6 Medicines for Children Research Network, c/o Somers Clinical Research Facility, Great Ormond Street Hospital, Great Ormond Street, London, WC1N 3JH, UK

**Keywords:** Activity theory, Chronic kidney disease, Common ground, Communities of practice, Ethnography, Long-term, Multi-disciplinary teams, Negotiation, Parents, Professionals

## Abstract

**Background:**

Limited negotiation around care decisions is believed to undermine collaborative working between parents of children with long-term conditions and professionals, but there is little evidence of how they actually negotiate their respective roles. Using chronic kidney disease as an exemplar this paper reports on a multi-method study of social interaction between multidisciplinary teams and parents as they shared clinical care.

**Methods:**

Phases 1 and 2: a telephone survey mapping multidisciplinary teams’ parent-educative activities, and qualitative interviews with 112 professionals (Clinical-psychologists, Dietitians, Doctors, Nurses, Play-specialists, Pharmacists, Therapists and Social-workers) exploring their accounts of parent-teaching in the 12 British children’s kidney units. Phase 3: six ethnographic case studies in two units involving observations of professional/parent interactions during shared-care, and individual interviews. We used an analytical framework based on concepts drawn from Communities of Practice and Activity Theory.

**Results:**

Professionals spoke of the challenge of explaining to each other how they are aware of parents’ understanding of clinical knowledge, and described three patterns of parent-educative activity that were common across MDTs: *Engaging parents in shared practice; Knowledge exchange and role negotiation*, and *Promoting common ground*. Over time, professionals had developed a shared repertoire of tools to support their negotiations with parents that helped them accomplish common ground during the practice of shared-care. We observed mutual engagement between professionals and parents where a common understanding of the joint enterprise of clinical caring was negotiated.

**Conclusions:**

For professionals, making implicit knowledge explicit is important as it can provide them with a language through which to articulate more clearly to each other what is the basis of their intuition-based hunches about parents’ support needs, and may help them to negotiate with parents and accelerate parents’ learning about shared caring. Our methodology and results are potentially transferrable to shared management of other conditions.

## Background

Evidence points to a rapid increase in the number of children and young people (children) with long term conditions such as chronic kidney disease stages 1–5 (CKD) since the 1960s [1]. The term CKD describes a complex set of long-term disorders with a wide range of primary causes and complications [2]. The total number of children with CKD is not accurately known, partly because early stage CKD produces few symptoms [3] and those it does produce are often non-specific. Therefore, CKD can be difficult to detect [4] and can go undiagnosed for some time. Progression towards stage 5 CKD (Table 1) is common, though the speed of this is quite variable; however, it is eventually fatal unless treated with a kidney transplant or dialysis.

**Table 1:** **The five stages of chronic kidney disease (CKD)**[5]

**Stage**	**Glomerular filtration rate**	**Description**	**Treatment stage**
1	90+	Normal kidney function but urine findings or structural abnormalities or genetic trait point to kidney disease	Observation, control of blood pressure.
2	60-89	Mildly reduced kidney function, and other findings (as for stage 1) point to kidney disease	Observation, control of blood pressure and risk factors.
3	30-59	Moderately reduced kidney function	Observation, control of blood pressure and risk factors.
4	15-29	Severely reduced kidney function	Planning for end stage kidney failure.
5	<15 or on dialysis	Very severe, or end stage kidney failure (sometimes called established renal failure)	Treatment choices (e.g. Dialysis or a kidney transplant).

Currently 870 UK children are receiving treatment for stage 5 CKD [6]. As children will have CKD for life and are at risk of long-term complications, early diagnosis and optimal management are essential [4,7].

Currently 870 UK children are receiving treatment for stage 5 CKD [6]. As children will have CKD for life and are at risk of long-term complications, early diagnosis and optimal management are essential [4,7].

Multidisciplinary teams (MDTs), with the support of parents, manage the care of children with CKD in the British network of 12 children’s kidney units. It is reported to be in children’s best interests to receive care for long-term conditions at home rather than in hospital whenever possible [4,7-9]so parents:*…perform the vast majority of care-giving, including tasks that are complex and demanding* ([4]:13). This means that professionals spend considerable time supporting parents as they learn to perform these tasks at home [10,11]. Individual parents have different learning needs and preferences but professionals do not necessarily know what these are when the child starts out on the ‘renal-journey’. However, if parents are uncertain about any aspects of clinical care-giving they may not maintain treatment regimens effectively or may fail to recognise the relevance of subtle clinical changes [3,12,13], so negative outcomes such as undetected urinary tract infections, damaged kidneys, hypertension, impaired kidney function, relapse of the condition, and transplant rejection may occur. These outcomes can lead to significant emotional, physical and financial costs for families [11], and they may have financial and policy implications for health services [4,14].

Few existing data relate to MDT management of childhood CKD, although a retrospective case-note review of 44 American children with renal insufficiency demonstrated better clinical outcomes for those managed in an MDT clinic compared to a general nephrology clinic, and multidisciplinary care was reported to improve outcomes of Canadian children with CKD [15,16]. However, interactions between MDTs and parents as they negotiate their respective roles when sharing children’s clinical care have received little research attention. Therefore, limited evidence exists to inform MDTs about the factors that are important in professional-parent interactions when parents are mastering the skills to incorporate clinical care into their day-to-day parenting roles. For MDTs, supporting parents to take on clinical responsibilities requires considerable time and resources; therefore, studying the way professionals and parents communicate about this is an important contribution to developing the evidence in order to augment effective practice. This will help experienced professionals determine how to individualise parent support from early in the trajectory, and inform the curricula for novice health professionals as they learn how to support parents.

Most evidence on parents’ experiences of living with children with long-term conditions draws on retrospective data from parents whose clinical care-giving practices were well established. This current evidence points to unresolved tensions between parents and professionals and a lack of negotiation around health-care decisions [14,17-20]. Furthermore, a recent Cochrane review of family-centred care for hospitalised children [21] highlights ineffective negotiations about roles of family members and staff that can cause resentment and communication difficulties.

Members of the current research team have previously undertaken studies that explored professionals’ and parents’ recall of how families learned about home-based CKD management. Specifically, participants described how parents learned to: collect and test urine; understand investigations; administer specialist diets, medications, gastrostomy or naso-gastric tube-feeds; manage peritoneal dialysis; monitor diet and fluids; recognise the importance of subtle clinical changes; record clinical observations; act on observations and results; and accurately communicate observations and actions to professionals [10,11,22]. Some parents reported that over time they coped with home-based clinical caring, but others reported negative emotional and physiological responses to the relentless clinical responsibilities [23,24].

Key limitations of previous studies, including our own, is that they: (i) focused on processes explored through retrospective qualitative interviews rather than direct observations of social interactions as professionals and parents shared clinical care; or (ii) did not all use conceptual frameworks to guide their enquiry; or (iii) used frameworks that highlighted issues such as adaptation by families, rather than social interactions [25]. Therefore, in this current study we used a mixed-methods design that involved a progressive focus beginning with a description and exploration of the broader context of CKD management in a national network of renal MDTs, to observing and exploring actual parent-professional interactions. We were particularly interested in the way professionals and parents used tools and artefacts (e.g. written information/documents and shared concepts/language) in the ‘practice’ of negotiating shared clinical caring, therefore, we adopted an analytical framework based on concepts drawn from Communities of Practice and Activity Theory. This approach [26] provided a robust framework to help us explore and discuss the way professionals and parents negotiate shared clinical care using tools and artefacts (hereafter referred to as tools).

Communities of practice, a conceptual perspective for helping professionals articulate the value of teaching and learning activities undertaken within communities comprise three dimensions of practice [27]. The first dimension is a *mutual engagement* of participants whereby individuals discover how to engage with each other, develop mutual relationships, establish who knows what about the common concern and negotiate meaning. Wenger defines negotiation of meaning as a productive process that denotes reaching an agreement between people and that negotiation conveys a flavour of continuous interaction, of gradual achievement, and give-and-take. The second dimension is the negotiation of a *joint enterprise* (the result of a set of shared tasks and a collective process of negotiation during which individuals fine-tune their practice and hold each other accountable to it). The third dimension is the emergence over time of a *shared repertoire* (including routines, tools and ways of addressing recurring problems). In communities of practice, experts’ knowledge is recognised to be tacit as well as explicit, with tacit aspects of knowledge often viewed as the most valuable as they consist of embodied expertise where a deep understanding of complex issues enables dynamic responses to context specific problems [28]. However, tacit knowledge is often difficult to make explicit, but individuals and groups with common interests and goals can produce useful tools to help explain tacit knowledge to each other. A domain of knowledge creates common ground, inspires individuals to participate, guides their learning and gives meaning to their actions.

Activity theory also concerns the study of practices and considers ‘knowing’ to be achieved through participation in practice [29]. Activity theory begins with the notion of ‘an ‘activity system’ of human ‘doing’ whereby ‘subject(s)’ (i.e. those working towards a shared aim such as parents of children with CKD and MDTs) work on an ‘object’ (i.e. the collective aim to achieve wellbeing for children with CKD); to do this ‘subject(s) use tools (such as written information and concepts). Therefore, activity theory focuses on subjects using tools to mediate negotiation.

For the purposes of this paper we used a methodological and conceptual framework that to our knowledge has not previously been used in this context, to shed new light on the ways professionals make their tacit knowledge explicit to each other and to parents in the process of negotiating shared clinical roles.

In summary, the literature supports the conclusion that the way professionals and parents share clinical care is not currently well understood and that research is needed which reports how professionals actually assist parents as they try to master clinical caring skills; the study reported here addresses this gap. The published protocol [30] can be found at: http://www.biomedcentral.com/1472-6963/12/33i.

## Methods

### Aim and objectives

The aim was to obtain a detailed understanding, rooted in the complexity and social context of practice, of the way MDTs support parents to undertake clinical care at home. The objectives were to:

1. Develop a descriptive profile of MDTs and their parent-educative activities

2. Explore professionals’ detailed accounts of the strategies they use when fulfilling these activities

3. Obtain a focussed and detailed understanding of professional/parent interactions as parents were embarking on delivering new clinical interventions at home

### Study setting

The 12 British children’s kidney units.

### Research design

**Figure 1:**
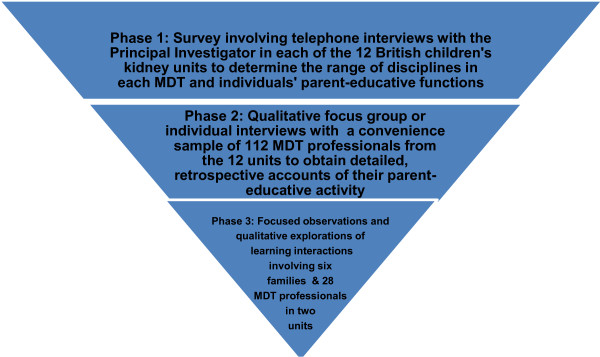
Study design.

To achieve breadth and depth of analysis we used a combination of quantitative and qualitative methods [31] in a three-phased design. Each phase formed a progressive focus on interactions during shared care encounters. The study overview is illustrated (Figure 1) and described in detail below.

### Phase I

We conducted a national survey of the MDT teams in the 12 children’s kidney units in England, Scotland and Wales to establish their strategies for supporting parents’ learning. We collected data through telephone interviews with local principal investigators (PIs). We developed a questionnaire designed to determine information such as: the number of professionals from different disciplines in each team, the information and skills individuals relay to parents, the teaching and support interventions they use, and treatment support needed by parents (e.g. post-transplant care, or management of haemo-dialysis, peritoneal dialysis, dietary restrictions, injections, naso-gastric tube feeding and complex medications). Additional questions asked whether individuals teach parents, reinforce information taught by colleagues, or teach and reinforce information.

The researcher administered the questionnaire during a booked telephone interview with the PI or a delegated colleague in each unit, at a mutually convenient date/time. The researcher entered the data provided into the questionnaires. Telephone interviews combined with administered questionnaires are an effective means of surveying busy clinicians [32], and can result in lower ‘missing-response' rates and less use of ‘don’t-know’ options than postal questionnaires. Data were managed using Excel to produce descriptive statistics, the derived profile of each unit informed Phase 2 data collection.

### Phase 2

**Figure 2:**
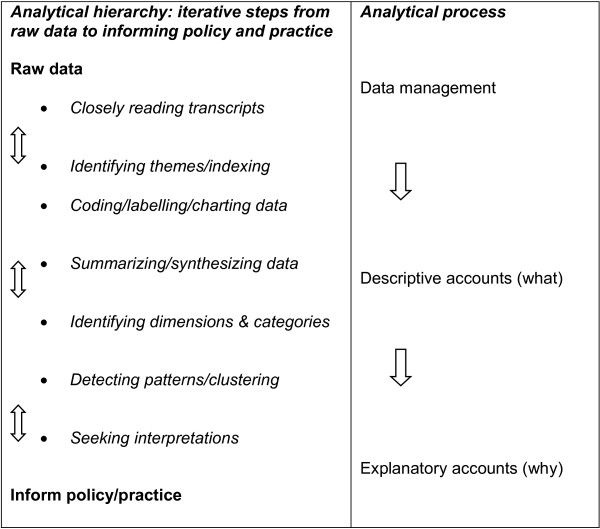
Analytical hierarchy and processes of framework.

The PI in each unit supplied the researcher with email addresses for all MDT members. Each MDT member received an email from the researcher containing a study information sheet, an invitation to participate and an expression of interest form. The invitation stated that for participants’ convenience, focus groups would be arranged to take place in the respective units, either before or after routine MDT meetings, but that interested professionals who were unavailable on the scheduled dates, or who preferred to take part in an individual interview would be offered alternative dates/times for individual interviews. Interviews were supported by a topic guide, and were digitally recorded, transcribed verbatim and anonymised. Data were analysed using Framework Analysis (Framework) (Figure 2).

Framework is a systematic and rigorous approach to qualitative data analysis [33-35], which draws on principles from different epistemological traditions within the social-science field. Transcripts were analysed through the five iterative Framework stages: (1) familiarization with the data; (2) identification of a theoretical framework; (3) indexing; (4) charting; and (5) mapping/interpretation.

Three researchers independently read and coded the first transcript (1), searching for patterns in the data, mapping connections and seeking explanations for patterns before comparing and discussing these until a consensus was reached. This resulted in the final framework (2) that was applied (3) to all transcripts by two researchers working independently with the remaining transcripts. Each coded transcript was (4) ‘lifted’ to a Microsoft Excel spread sheet for charting where quotations were labelled for retrieval during reporting. In stages (4) and (5) data from disciplinary datasets were coded across and between the 12 units. As data management proceeded, emerging themes supplemented interview topics; this iterative process involved moving backwards and forwards between the Framework stages [33,35]. This helped us identify new lines of enquiry to pursue during on-going data collection and analysis. Constant comparison of data within and between themes opened up meaning in the text until no new themes emerged. To ensure trustworthiness and credibility, reduce potential bias and enhance theoretical sensitivity we incorporated reflexivity into the data management process regularly considering whether analysis might have been compromised in favour of our own preconceived ideas. Selected Phase 2 data are presented and discussed below in the Results section, but an additional file also presents further Phase 2 data for information [see Additional file 1].

### Phase 3

#### Sample selection and recruitment

Using an ethnographic approach involving the systematic, detailed observation of behaviours and talk [36], we undertook six focussed, observational case-studies in two of the children’s kidney units. To protect individuals’ identity the two units are not named, but they were selected by the study Steering Group after scrutinising and discussing Phase 1 results. The two units were selected on the basis that they were the most likely units to yield 4–6 index cases (each one the focus of a case-study) within the timescale, that met our purposive sampling criteria, and that would allow us to achieve maximum sampling variation based on children’s age, sex, ethnicity and the type of new, clinical home-based care-giving that parents were embarking on. Each case study lasted six months. Snowball sampling^a^ initially identified the professionals involved in management of index cases; convenience sampling was then used when the researcher encountered other relevant professionals.

#### Data collection

As the two selected units had participated in Phase 1 and 2, and the Phase 3 researcher (RN) had spent time meeting with MDT members before Phase 3 data collection commenced, professionals were already aware of the study. When both parents were involved in learning clinical skills and knowledge, informed consent was sought from each, and the assent of children involved in any observed events was sought each time if they were capable of providing this. We were aware of the possibility that some participants may have felt they were being judged on their performance, therefore, RN regularly reinforced assurances given during the recruitment stage that the study was not ‘testing’ knowledge or ‘judging’ teaching, parenting or professional care-giving skills. We viewed consent as a process rather than a single event meaning it was regularly reviewed with patients, parent and professional participants during Phase 3. Participants were given the opportunity to signal using a coloured card if they wanted the researcher to leave an observed situation. Support from a Clinical Psychologist on the research team was available for anyone who became distressed by participating in the study. Neither of these measures were utilised by any participants, though the researcher decided to leave one observed situation when the child was becoming distressed during a clinical care-giving task.

Professional/parent interactions were explored using a combination of data collection methods (Table 2) as parents learned new skills and knowledge.

**Table 2:** Data collection in Phase 3

**Method used**	**Context of data collection**
86 observations of parent/professional interactions	• In wards, outpatient departments and families’ homes during planned and ad-hoc interactions
	• Verbatim field notes (recording behaviours, context, time, personnel and environment).
41 individual semi-structured interviews with family members and professionals following selected observations	• Exploring participants’ views about how parents were supported to deliver home-based clinical care, including the effectiveness of the observed interactions
	• Interviews lasted 20 – 65 minutes, were digitally recorded and transcribed verbatim.
Selected case-note reviews	• Obtained relevant background information and noted how professionals documented interactions with parents.
Reviews of documents	• Used by professionals and parents to support their interactions
	• Used by parents to record home-based clinical caring

As data collection proceeded, the inquiry progressively focused on specific research questions, for parents for example, this could include interview questions which built on what professionals told us in Phase 1 and 2 and related to specific observations/interviews in Phase 3; these included questions such as:

•What new information and skills have you recently had to learn?

•What helped you to learn?

•Has there been anything you have found difficult to learn?

•Was there anything about the way you were taught that you found less helpful?

•Was there anything you would like to have been done differently?

Questions for professionals included:

•Can you tell me what you have been helping these parents to learn?

•How do you feel these parents are learning?

•What did you think worked about the teaching session that just took place?

•Was there anything you would have done differently?

•Did you make any changes to how you would normally demonstrate that?

•What made you decide to explain that in the way you did*?*

•How did you decide how much information to give this parent?

•How did you decide what order to teach things in?

This approach allowed for strategic data collection during observations, interviews, case-note and document reviews so that answers to questions could be pursued more effectively and tested against existing data and literature. Data analysis involved Framework (as in Phase 1). Study approval was obtained from North-West 3 Research Ethics Committee (REC) (reference: 09/H1002/92), the University REC, and the participating NHS Trusts.

## Results

### Phase 1

Table 3 reports on the professionals from each discipline across the 12 units who were reported to be involved in teaching parents and/or reinforcing colleagues’ teaching.

**Table 3:** Phase 1, number of staff across the 12 MDTs involved in teaching parents and reinforcing colleagues’ teaching

**Discipline**	**Teaching only**	**Reinforcing only**	**Teaching &reinforcing**	**No teaching or reinforcing**
**Consultant paediatric Nephrologists**	0	25	39	0
**Junior doctors**	0	12	28	7
**Renal Specialist nurses**	0	0	48.2	0
**Ward nurses**	0	36	284	0
**Haemodialysis nurses**	0	7	125	0
**Peritoneal dialysis nurses**	0	25	220	0
**Health care assistants**	6	5	11	17
**Dietitians**	0	0	19	0
**Counsellor/Therapist**	0	0	1	0
**Clinical Psychologist**	0	6.6	3	0
**Pharmacist**	1	2	5	3
**Play-specialist**	1	0	15	0
**Social-worker**	1	3	3	2

### Phase 2

An opportunistic sample of 115 health professionals expressed interest and 112 participated in group (n = 13) or individual (n = 7) focused interviews (three professionals who expressed interest were subsequently unavailable during the data collection period so did not participate). The final sample comprised: seven Clinical Psychologists, nine Dieticians, 30 Doctors (28 Consultant Paediatric Nephrologists; two Registrars), 48 Nurses (included the roles of Specialist Nurse, Nurse Consultant, Nurse Specialist, Clinical Nurse Specialist, Associate Nurse Specialist, Advanced Nurse Practitioner, Staff Nurse, Senior Staff Nurse, Junior Sister, Sister, Matron, Ward Manager, Research Nurse, Clinic Nurse, Community Nurse, Ward Nurse, Nurse working in haemo dialysis, peritoneal dialysis or transplant), three Pharmacists, seven Play-Specialists, six Social-Workers, and two Therapists).

Data presented here are drawn from these interviews. Participants spoke at length about their experiences and touched upon a wide variety of topics in describing the way they helped parents learn to administer home-based clinical care. Professionals discussed how they delegated day-to-day clinical responsibilities to parents. Shared care therefore, involved frequent interactions between a large number of professionals (representing all disciplines in the respective MDTs) and the children’s mothers and/or fathers.

Our analysis identified three patterns of parent-educative activity that were common across all MDTs: (i) *Engaging parents in shared practice, (ii) Knowledge exchange and role negotiation*, and (iii) *Promoting common ground.* Although there is some overlap between these patterns, for clarity they are presented sequentially below. Professionals’ accounts indicate that they move backwards and forwards between activities according to the child’s clinical status and parents’ perceived support needs.

#### Engaging parents in shared practice

For professionals, an essential part of their role was engaging parents in a mutual process of discussion about their child’s clinical needs, and the management of these needs, before parents collaborated in shared caring. Professionals spoke of sharing discipline-specific knowledge with each other and with parents, and explained how they drew upon these interactions to determine the knowledge and skills parents needed to acquire in order to share their child’s care. Central to this was the need for professionals to establish a good ‘working relationship’ with parents:

If you get it [relationship with parents] wrong in the first few hours, you have many problems really (Nurse_68).

However, in the absence of a standardised tool to assess parents’ learning needs and preferences, professionals often spoke of relying on their own and each other’s’ tacit knowledge to help them determine how to pitch communication, as this focus-group discussion illustrates:

It's [knowing how to judge parents’ understanding] just intuition really…, based on parents’ body-language, verbal-language, the words they're using. And I couldn't sort of put into words how you do that [Nurse_5]

… mmmh, you're saying something and you can see it literally going on in their head, can't you? Or you can see whether they're actually understanding…[Nurse_ 7]

I agree very much that it's intuition that allows you to initially try and decide what level you want to pitch things…(Doctor_37).

This discussion was typical of the concerns expressed by professionals about the challenge of making implicit (or tacit) knowledge explicit during a process of mutual engagement; both by articulating to each other the basis for their intuitive hunches about parents’ support needs but also by making their professional knowledge explicit to parents in a way that was meaningful to the parents.

#### Knowledge exchange and role negotiation within shared care

Professionals frequently spoke of knowledge exchange within the MDT and between MDT members and parents as they negotiated their respective roles. Types of knowledge exchanged included specialist clinical knowledge and day-to-day practice knowledge. A particular challenge that professionals identified was the fact that individual parents’ situations and possible responses to situations vary from one to the next, and from one day to the next.

However, despite this variation professionals believed that they had a responsibility to negotiate with parents the best way to manage their own child’s clinical care, and to be clear about the on-going home based clinical responsibilities that were likely to be necessary:

We are all the time negotiating with each other in the MDT and with them [parents] what we need to do together to support the child (Clinical Psychologist_7)

In this way professionals appeared to be describing the negotiation of a joint enterprise with each other where the MDT served a very important function as a sounding-board, thus enabling professionals to rehearse with colleagues what information to convey to parents, and how best to do this:

…before agreeing on a united care-plan or list of options to discuss with parents (Doctor_72)

Team working was described as an essential part of knowledge exchange and role negotiation with parents. Professionals talked of the importance of aligning the team’s goal of achieving optimum clinical management with the needs and preferences of the child and parents. In doing this, professionals spoke of strategies they had developed through experience of caring for children; so for example, several participants described using concepts such as a staged approach to sharing specialist clinical knowledge within the MDT, and understanding how parents might manage the child’s care at home. This staged approach also helped professionals to provide parents with a contingency plan, as the following data illustrate:

…teaching them very slowly and not letting them rush…they’ve really got to know what might happen if you do this, if you do that…(Dietitian_6).

#### Promoting common ground during shared care

In keeping with the MDT goal of seeking to collaborate effectively with parents through role negotiation and knowledge exchange, professionals aspired to promote common ground in the practice of shared care. Common ground is the sum of mutual, common, or joint knowledge, beliefs, and suppositions. The need for common ground operated at two levels: (i) a common understanding between professionals about the level of parental understanding; (ii) and on the basis of trying to establish common ground with parents this fed back into the former as professionals drew on their tacit knowledge to understand and explicate to each other how parents were managing shared clinical care.

We have a weekly psychosocial meeting where they [families] would be discussed at an early stage….Where there was sort of important things going on they [the important things]would be brought to that meeting …without the MDT it [management] simply doesn’t work (Social worker_18)

Professionals described the way they had over time, developed a shared repertoire of intradisciplinary and interdisciplinary tools to use as resources when engaging parents in their child’s clinical care. From an activity theory perspective tools were used to solidify ideas and as mechanisms for helping to create common ground between individuals working together. These tools include ‘tricks of the trade’ such as checking whether they had explained themselves ‘properly’ to parents as the following data illustrate:

…we don’t review learning progress formally as if teaching health-professionals… but we’d probably do that informally every time we sit in clinic or have a discussion with them (Dietician_99)

The shared repertoire of tools also included words, phrases, metaphors, routines, dolls, stories or concepts. For example, discussions with parents about their child’s clinical care were often supported by using metaphors such as:

How the kidney does a lot of work and is made of a whole lot of little factories (Doctor_113).

Furthermore, because many kidney conditions have no associated physical findings professionals often tried to promote common ground with parents by explaining disease processes using diagrams, blood tests or scan-results:

…explaining about things happening inside [the body] as often they’re not terribly obvious on the outside (Doctor_103)

In summary, professionals described using tools within practices of mutual engagement, negotiation of a joint enterprise and development of a shared repertoire with each other in the individual and shared endeavour of supporting parents. Looking at practice through the lens of communities of practice and activity theory led us to view collaboration and negotiation as being essentially about establishing common ground. The concept of common ground has been used extensively in the field of computer supported collaborative work and the practice-based organisational literature as a means of understanding how workers cooperate to achieve a common goal. For example, Bechky [37] described the process by which understanding between engineers, technicians and assemblers in the field of computer supported collaborative work is transformed across occupational communities, generating richer understandings of the production process within the organisation. Much of the work on common ground draws on theoretical influences including communities of practice and activity theory, but going beyond this. However, to the best of our knowledge, this approach has not been applied to collaborative working in the context of shared clinical care. An additional file presents Phase 2 data in more detail [see Additional file 1].

### Phase 3

Eighteen family members (6 children, 6 mothers, 4 fathers and 2 grandparents) (Table 4), and 28 professionals (4 Dieticians, 9 Doctors, 10 Nurses, 1 Pharmacist, 1 Play-worker, 1 Social-worker, 2 Therapists) participated.

**Table 4:** Characteristics of participating families

**Families’ study identifier**	**Child’s age**	**Child’s sex**	**Child’s ethnicity**	**New clinical responsibilities by parent(s)**
1	8	Boy	White British	Home dialysis.
				Parents had previous experience of home-based care-giving.
2	11	Girl	White British	Home dialysis.
				Previous experience of home-based clinical care-giving.
3	12	Girl	White British	Dietary restrictions, preparing for home dialysis.
				Previous experience of home-based clinical care-giving.
4	15	Girl	South Asian	Understanding new condition, medication, diet, home dialysis.
				Little experience of home-based clinical care-giving.
5	3	Girl	White British	Post transplant (e.g. fluids, medication, diet, NG tube feeds).
				Previous experience of home-based clinical care-giving.
6	5 months	Boy	White British	Understanding new diagnosis, fluid management, NG tube. Parents had little experience of home-based clinical care-giving.

Parents recruited were embarking on new clinical caring task/s at home, these included:

•Administering complex medications, dietary supplements, gastrostomy or naso-gastric tube feeds

•Setting up/running home dialysis

•Monitoring diet and fluids

•Recognising subtle clinical changes

•Recording clinical observations

•Acting on results

•Accurately communicating observations/actions to professionals

**Figure 3:**
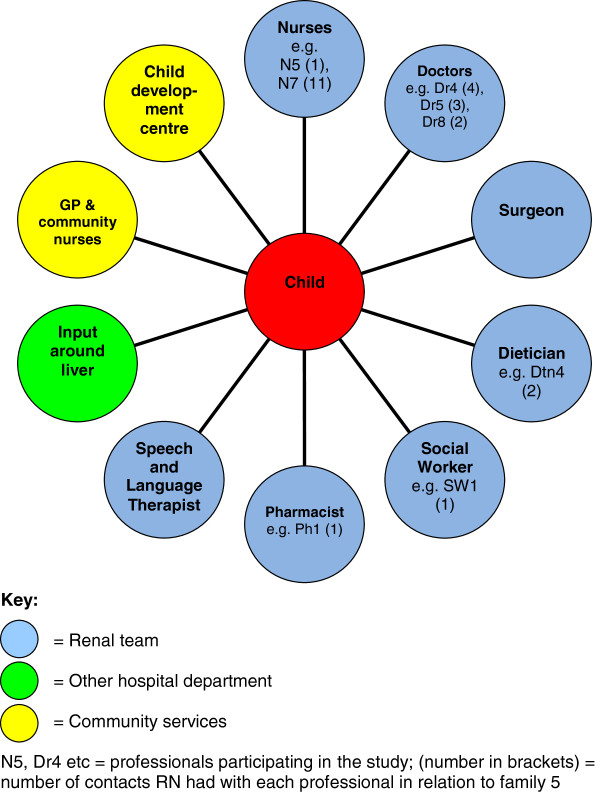
Example of a case-study family-professional network.

Figure 3 provides an example of the range of professionals involved in supporting one case-study family.

### Accomplishing common ground in CKD shared care

In this section, brief focussed descriptions illustrate the central theme arising in professionals’ and parents’ experiences. The themed description of results created a conceptualisation of shared caring that we defined as *Accomplishing common ground in CKD shared care*. This means that unlike data reported in earlier studies where collaboration in relation to care-decisions was lacking, our ethnographic data consistently demonstrates parents and professionals sharing children’s clinical care. Focussing on this shared activity has offered new insights into parent/professional collaborations as they negotiated their respective roles during shared clinical caring.

#### Setting

From early in the trajectory parents seemed to cautiously accept day-to-day responsibility for clinical care as part of their everyday parenting role. Professionals were often observed acknowledging parents’ expert knowledge of their child when facilitating parents’ clinical role development. In parallel with this, we observed parents trying to meet their child’s clinical needs in collaboration with professionals while also coping with the everyday emotional and practical challenges of parenting. We frequently observed professionals sharing a wide range of tools with parents as part of shared care; these included blood results, leaflets, diagrams, and dolls and shared concepts such as teamwork and competence. The following vignettes are based on the case studies and use field note data *(presented in italics)* and discussion to help illuminate the concept of *Accomplishing common ground*.

### Vignette 1

In the following field note excerpts, we depict two consecutive clinic consultations between a doctor, a pre-school patient (whose CKD had been managed by the MDT for several months but who had recently received a kidney transplant), and the child’s mother. We observed the doctor: (i) engaging the mother in discussion about the child’s medications, and the blood results that indicate how the child’s transplanted kidney is functioning; (ii) acknowledging the challenge of ensuring the child takes prescribed medications (iii) reassuring the mother that the medicine regimen will become less complex over time; and (iv) encouraging the mother to ask questions. We also observe the mother using her knowledge of clinical terminology to discuss blood results with the doctor.

The child and mother in this scenario had moved from the ward to the patient hotel^b^ the day before the first consultation. The doctor uses the consultation to review the child’s treatment regimen. First, we see a process of mutual engagement where the doctor exchanges pleasantries with the child and the mother (who is holding a medicine sheet listing and explaining the child’s medications; she had previously received the sheet from the pharmacist and had been using it in the patient hotel when preparing for making the transition home). The doctor then casually steers the conversation towards what appeared to be the primary objective of finding out how the mother was managing the child’s treatments. In this dialogue, we see the mother and doctor mutually engaged in negotiation. According to the communities of practice concept, being included in what matters (i.e. discussion around the child’s clinical care) is a requirement for mutual engagement, but this can be very subtle, so it can be difficult to distinguish between the value of the specific information exchanged and, the personal exchanges that are woven into the discussion.

Consultation 1:

Doctor: So two weeks today [since transplant]. She’s done really well

Mother: Her creatinine is quite stable

Doctor: How are the meds going?

Mother: I’m starting to get them into my head

Doctor: What are the most important ones?

The mother correctly names two medicines and receives praise from the doctor. The doctor looks at the mother’s medicine sheet and continues to discuss the medications, reinforcing the function of each one (e.g. ‘This is for her blood-pressure, this is for her liver and bones’) and reassures the mother that:

She’ll be on about a third of these at 6 months [post-transplant]. At the beginning, it’s very mind blowing, you’re doing really well. If all else fails, make sure you give her [name of drug]…so if you’re having a bad hair day!

Through this on-going discussion, we see specific information exchanges and personal exchanges being woven into the conversation with the tool [the medicine sheet] being used to negotiate common ground. This is entirely consistent with the insight derived from activity theory whereby tools are central to the process of negotiation, and we begin to see the second characteristic of a community of practice emerging through the negotiation of a joint enterprise between the mother and the doctor. Within the structure of the healthcare context we see the doctor (who has overall responsibility for the child’s CKD care) and the mother (who has day-to-day clinical responsibility as well as a vested emotional interest in her child) confirming the child’s response to treatment. The mother’s comments on creatinine also position her as knowledgeable. Both the doctor and the mother also appear to be making the practice of joint enterprise amenable for themselves, so their conversation, with its mixture of acquiescence and assertion, is a complex, jointly negotiated response to what they understand to be their situation.

Having confirmed that the mother has a good understanding of the medication regimen, the doctor directs discussion towards the blood results, in the knowledge that the mother has already highlighted that her child’s creatinine has been stable since she last saw this doctor. The doctor then turns to the desktop computer to find the child’s blood results on the laboratory database.

Doctor: Blood results are lovely.

Mother: Is that the creatinine?

Doctor: It’s beautiful, it’s 16.

The doctor shows the mother the graph of the child’s creatinine levels on the screen

Doctor: It’s 16, I can’t argue with that. Her calcium is good, phosphate is good, magnesium is good.

Mother: What about her urea?

Doctor: That’s 1

Mother: Where would you expect it to be?

Doctor: Usually a bit higher. Albumin is good. I can’t ask you to do anything else; you are doing a fantastic job. If you have any questions during the night, don’t think they are ‘stupid’, just write them down and bring them to clinic

The doctor’s reassurance and encouragement is backed up by advising that if the mother has any questions during the night she should write them down and ask them in clinic, even if to the mother they ‘seem stupid’. The doctor provides guidance on what to do with the child’s medication regimen ‘if all else fails’ and ‘if she is having a hair-bad day’, meaning that the doctor acknowledges that clinical caring can be difficult and that things don’t always go to plan. However, the doctor appears to be reassuring the mother that she would not be judged as ‘irresponsible’ if there is a day she finds it very difficult to give the child all the medications, but that there is one drug she must give at all costs.

The doctor also carefully explains the different blood results, indicating whether or not they are within the expected range. In this observation we see a co-operative process whereby tools (blood results and the medicine sheet) are used as a shared repertoire between the doctor and mother as a resource for negotiation of meaning around their shared goal (the child’s optimum clinical outcomes). We also see the doctor and mother holding each other accountable to the achievement of optimum clinical outcomes for the child. Using a framework of communities of practice, activity theory and common ground has allowed us to explore the way these tools enabled the doctor and mother to engage in a mutual process of seeking and offering clarification around the child’s clinical care. The tools required the mother as well as the doctor to have a competent understanding of the clinical problem, and sufficient specialised vocabulary with which to exchange ideas.

In the second consultation two weeks later, the child, mother and doctor are in clinic together again.

#### Consultation 2

When discussing the child’s progress the doctor turns the computer screen so the mother can see the ‘creatinine’ graph, the doctor’s bleep sounds so the doctor makes a phone-call during which the mother offers an unsolicited explanation to the researcher on the significance of the graph. The doctor turns her attention back to the mother and it would appear that the doctor has confidence in the mother’s capacity to understand the important connection between medications, diet and blood results as she asks no further questions. Instead, the mother asks what the child’s sodium levels are and when told it is ‘just right today’ the mother suggests she should ‘stick with 3 sodium chlorides, 3 times a day’?, and the doctor agrees.

It appears from this vignette that the doctor and mother had accomplished common ground where they were both satisfied that the mother had a good understanding of her child’s medicines, diet and blood results; they used tools extensively during the consultation and demonstrated how they both negotiated meaning using their shared repertoire. Indeed the mother commented when later interviewed that the way the pharmacist had tailored the medicine sheet for her (it used layman’s terms and included diagrams), and the way the pharmacist had supported the use of the medicine sheet with verbal explanations, made it very easy for her [the mother] to understand the medications. In a later interview, another doctor who had also been involved in managing the child’s care before and since the kidney transplant, when asked how they felt this mother was managing the child’s new treatment regimen said:

Oh, I think she has done fine with learning medications… I don’t think I’ve ever been aware that she’s got medication doses wrong, or anything like that…she probably takes a lot of pride in how careful she is.

This example shows the mother as being very competent and able to use technical language and read the computer graphs, and the doctors being very confident in the mother’s ability to add a new set of clinical responsibilities to her existing repertoire of clinical skills within her parenting role.

Because of a shared assumption that children’s interest are best served by them being in their normal environment whenever possible, it was implicit in all the interactions we observed that there was little scope for negotiation about whether or not parents would take-on home-based clinical responsibilities. However, we often observed negotiations between professionals and parents about what skills parents actually needed to develop and what their needs and preferences were when developing these skills. These issues are explored and discussed in the following vignette; in particular, we saw negotiations concerning the practical challenges of clinical caring in the home where parents have primary responsibility for their child’s wellbeing.

### Vignette 2

In the following field note, we see a nurse, a small child with CKD, and the child’s mother and father together in a room on a ward. In this vignette, the child was newly diagnosed and the parents are novices in the shared clinical caring role. The nurse, aware that the parents are new to this role is: (i) engaging them in helping her replace their child’s nasogastric tube; (ii) reassuring the parents and encouraging them to cuddle the child (a role they would usually engage in as parents); (iii) encouraging the parents to imagine that inserting the tube might be done without ‘hurting’ their child; and finally (iv) in helping the parents to see that they might reinsert the tube when the child was being cared for at home:

Father lays the child on the plinth [to allow the nurse access to change the tube]. Nurse removes the old tube saying:

‘Take him up and give him a cuddle’ [Father does this]. ‘It doesn’t hurt taking it out; it’s just a weird sensation’.

Father lays the child down again and the nurse inserts a new tube. The child is crying a lot, the father is holding the child, while the mother and nurse talk to the child to try and soothe him [the child].

Mother to father: ‘You hold his head and I’ll hold it [the tube]’

Nurse finishes: ‘There, have a cuddle’. Mother picks up the child and cuddles him

Nurse to parents: ‘Thanks for helping’

Mother: ‘It’s what we do at home, one holds [the child] and the other does [what care is needed].’

Nurse: ‘That’s teamwork. Are you interested in learning how to do it?’ [Insert the tube]

Mother: ‘Yes, if he’s going to need it in the long-term?’ [that is, if it will be necessary for more than a few weeks]

Nurse: ‘It’s likely. He produces so much urine; he will need the overnight feed [To replace fluid lost when passing urine].

From a communities of practice perspective this vignette demonstrates how delegation of the task [inserting the tube] from nurse to parents involves a process of negotiation and re-negotiation, whereby the nurse enables parental engagement in the shared practice (i.e. insertion of the child’s tube), and explicitly recognises the parents’ unique relationship with their child as being his emotional and caring support. Crucially, the nurse reassures the parents that the child is not ‘hurt’ and invites the parents to imagine engaging in this role at home, before asking them if they would be ‘interested in learning how to do it’. The answer is “yes” if there is a ‘need’. Furthermore, the nurse’s comment ‘thanks for helping’ is an acknowledgement of the parents’ expertise; the parents confirm that they are used to sharing the child’s care at home, which then seems to prompt the nurse to invite them to expand their clinical skills. The nurse reads from the parents’ response that they are coping and not being too distressed by the child’s distress, so are perhaps ready to take on more clinical responsibilities.

It is implicit here from the community of practice perspective, that the MDT and the parents share a joint enterprise of caring effectively for the child’s clinical needs at home and use a process of ‘give and take’ to achieve this. Thus, we argue that the practice of delegation of ‘skilled’ care from MDT to parents involves negotiation, but this negotiation works because of the implicit, shared understanding of the joint enterprise of meeting the child’s needs. Furthermore, through highlighting to the parents the connection between the child’s increased fluid intake, the need for replacement fluids via the nasogastric tube and the pragmatic benefit for the family of parents’ learning to re-insert the tube at home [instead of returning to hospital each time it needed to be re-inserted], the nurse and parents are engaged in using part of the shared repertoire of the community [clinical and practical knowledge] as a resource for the negotiation of meaning.

In an interview about a month after this consultation, the nurse discusses the challenge of working with parents when they first hear their child needs a nasogastric tube. The nurse recognises that parents go through ‘a learning process’ of not believing and/or accepting that their child needs a tube, learning by ‘trial and error’ and then accepting the tube is needed. As a result, the nurse proposes that she needs to ensure parents have time to accept that their child will need on-going clinical care at home; and that this process of learning and acceptance cannot be rushed:

I’ve learned through the years that you can’t force this process it’s something I think parents have to just slowly get to themselves; our instinct as parents is to be able to feed our children and look after them well and one of the things that you do by looking after your child well is to feed them and give them fluids, I think that’s actually a deep rooted instinct and that first of all having a nasogastric tube is a huge step for parents to overcome. I often hear parents talk about their sense of failure in terms of ‘…I couldn’t do this right [nourish their child] because otherwise they wouldn’t need that tube’

This helps to explain why the nurse appeared to be so careful in her negotiations with the parents.

In summary, these two vignettes have highlighted the way MDTs and parents use tools to engage around the common problem of managing children’s CKD. An additional file shows further examples of tools we observed being used [see Additional file 2]. The vignettes we have presented in this paper help to demonstrate how professionals draw on parents’ expert knowledge of and relationship with their child, thereby negotiating a joint enterprise (optimum management of childhood CKD), and accomplishing common ground through development of a shared repertoire that supports parents as they take on new or additional clinical responsibilities.

## Discussion

Policies and guidance acknowledge that parents of children with CKD perform the vast majority of complex and demanding clinical care at home, and that the paediatric renal MDT is a focus for parents to seek specialist support for this aspect of their parenting role [4,38]. The fact that parents develop considerable expertise in managing a range of children’s long-term conditions is widely acknowledged, but research has consistently indicated that parents believe their expertise is not valued by health professionals, and tension and conflict between parents and professionals are often reported [17].

In this paper, we have developed an account of parent-professional practice using a conceptual and methodological framework not previously used in this context to help illuminate the process of shared clinical caring. Through progressively focussing on the research topic whereby we moved from describing, exploring and discussing the broader context of CKD management in a national network of renal MDTs, to observing and exploring parent-professional interactions in particular cases, we have provided new insights into shared clinical caring activities. Using participant observation combined with interviews, case-note reviews and field-notes enabled us to explore professional/parent interactions and the way tools were used to accomplish common ground within the three dimensions of practice (mutual engagement, joint enterprise and shared repertoire) [27,29].

Professionals’ primary focus was on diagnosis and treatment of the child’s clinical problem, while parents’ primary focus was on their child’s overall wellbeing, and the role(s) they themselves needed to adopt to share their child’s clinical care with the MDT. Because many aspects of the clinical role were delegated to parents to undertake at home, there was a shared assumption that parents would lead the day-to-day clinical role, and therefore, that parents need MDT support to help them deal with the associated practical and emotional challenges.

Our data have demonstrated both similarity to and divergence from the literature concerning family management of long-term conditions. This study’s primary contribution is in shifting the focus away from that reported in the literature that investigated the work associated with family management, information needs and parents’ roles in a range of long-term conditions (but rarely including CKD); notably these studies looked broadly and usually retrospectively at parents’ management activity [14,17,20,39-41]. Furthermore, a Cochrane review [21] that highlights ineffective negotiations about roles in hospital settings calls for researchers to identify effective models of care which may ameliorate such communication breakdowns. The current study addresses this issue; to the best of our knowledge it is the first to elicit data, foster longitudinal and focussed insights, and report on the actual parent-professional experience of shared care and negotiation between parents and renal-MDTs. These insights are generally not as accessible through other research approaches such as those reported in the literature (e.g. quantitative methods or retrospective qualitative accounts).

Contrary to reports in the literature (e.g. [17,21]) we did not observe or elicit accounts of tension or conflict between parents and professionals. Where tensions were occasionally evident in our data this was either within families or within MDTs, but not between professionals and parents. We have reported and discussed this issue elsewhere [42].

As is usual in observational research the Phase 3 sample was small and may not represent all phenomena that affect parents using child-health services, so we do not claim that our findings are representative of all parents. Therefore, our findings may be an anomaly of this particular population, or may have been influenced by the presence of the researcher [36], so further research is needed to investigate these issues.

Additionally, the findings may differ from other reports because previous studies tended to rely on respondents’ retrospective accounts, or the research questions posed may have focused only on participants’ difficulties and negative experiences whilst our approach was prospective and asked a range of closed and open-ended questions about participants’ experiences.

Moreover, recent research into family-management of long-term conditions of childhood [43], including our own prior research around CKD management [11,41] highlights a gap in our understanding of the way health-care professionals provide specialist support, and the ways parents learn to master treatment regimens and fit them into their everyday life [43]. Our study also addresses this gap through a methodological approach that is novel in the field of shared clinical care; therefore, we argue that this paper makes a methodological contribution to knowledge in this area.

Previous studies in this area mostly drew on data collected from parents whose care-giving practices were already well established, and who had consequently developed their own unique management styles (e.g. [44]). There is little prior evidence of observational research that captures parent/professional interactions when parents are actually learning to administer clinical care to their child with CKD, or that uses the progressively focused approach we adopted in this study.

The professional participants in this study were experts in clinical care of children with CKD, and in supporting parents in the shared caring role. However, novice practitioners will need explicit guidance for this aspect of their role so our data can be used to inform curricula for the education of undergraduate practitioners, thereby helping them to develop the skills to promote parents’ shared clinical caring skills from early in the child’s condition trajectory.

The key theoretical contribution of this paper focusses on the idea that professionals can find it challenging to make tacit knowledge explicit to each other and to parents when engaging parents in shared practice. Firstly, it identifies the existence of mutual engagement during interactions between professionals and parents around the shared concern of achieving optimum clinical management and wellbeing of each child with CKD. In line with Wenger’s definition of negotiation, our data and discussion illustrate continuous interaction between parents and professionals whereby gradual achievement was evidenced (by parents as they mastered clinical skills and knowledge, and by professionals as they acquired understanding about parents’ individual needs and preferences), and of ‘give-and-take’ between parents and professionals during the process of mutual engagement. Next, our theoretical contribution demonstrates a collective process of joint enterprise that was the result of a set of shared tasks, and a collective process of negotiation during which parents and professionals fine-tuned their practice and were observed to hold each other accountable to the achievement of optimum clinical outcomes for the child; this reflects the full complexity of mutual engagement. Finally, it introduces the idea of *accomplishing common ground* whereby professionals develop and use a shared repertoire of tools for negotiating meaning with each other and parents about children’s clinical caring needs.

Whereas the adaptive practice model [25] seems to imply that the only important communication in child-health care is unidirectional (i.e. from health professional to parent) and that professionals have little to learn from parents, our conceptual framework has illuminated a two-way process of communication where parents’ expertise is recognised and valued by professionals, and parents learn to communicate with professionals about shared caring. Although there is now a growing awareness in the health and social care field of the potential of Communities of Practice [27] and Activity Theory [29] to help address complex health care situations, to our knowledge no other reports use these concepts in the way we have used them to explore social interaction between MDTs and parents as they share children’s clinical care. Therefore, we argue that our contribution adds to this important field by extending the evidence base.

Previous ethnographic studies vividly demonstrate the insights that observational techniques can engender, and confirm the conclusion that they may be better suited than other methods to examining complex interventions in child-health contexts [45,46]. However, ethnographic studies that focus on parents’ experiences are historically underutilised in child-health settings, in particular with research that seeks to understand the complex intervention [47] of shared caring in CKD management. A promising direction for future research would be to observe and analyse parent-professional interactions later in the trajectory when parents’ care-giving practices are established to determine whether the characteristics of shared caring change over time.

A body of evidence is emerging about a positive relationship between MDT support and clinical outcomes [15,16] although this does not include qualitative studies to help interpret these results within the context of MDT/parent interactions. Our study contributes to this evidence-base by producing new insights into the way parents and MDTs embark on shared caring. One key advantage of MDT care as described in this paper is that an MDT gives multiple opportunities to interact with parents (using a combination of uni-disciplinary and multi-disciplinary tools), and feed back into the team their level of understanding as each professional engages with parents about different elements of their child’s care using different tools.

Previous studies mostly focussed on parent support provided by doctors and nurses, with little evidence of other disciplines’ contributions although multi-agency working has been explored in the context of complex health needs [48]. However, we believe the study reported here is the first to focus on the way a national network of MDTs facilitates parents’ clinical role development, and the first to use a longitudinal, mixed-methods design to explore ‘live’ parent/professional communications. This study has started defining the vital ingredients of the complex-intervention [47] of shared renal care, future research that builds on this study could involve development and testing of a multidisciplinary assessment tool to determine parents’ individual support needs and preferences as they embark on the process of shared clinical caring.

Researchers and clinicians are sometimes reported to be working in isolation from each other thereby compromising coordinated attempts to develop a knowledge base [49]; a strength of this study is that it represents a multidisciplinary collaboration involving researchers, clinicians and parent advisors. Our results could also be transferred to other clinical contexts where parents undertake similar types of complex, home-based clinical care (e.g. cancer, rheumatology or cystic fibrosis services).

## Conclusions

In summary, through progressively focussing on MDT-parent interactions using a mixed-methods approach, and by employing a conceptual framework that explicitly acknowledges the value of tools within the practices of mutual engagement, joint enterprise and shared repertoire, we can offer new insights into the process of shared clinical caring for childhood conditions such as CKD. These insights highlight the significance of two key aspects of MDT-parent interactions and the way they contribute to the process of accomplishing common ground within MDTs and between professionals and parents. The first key aspect of social interaction is the way tools act as a medium for shared caring; and second is the fact that negotiation and renegotiation of roles is a two-way process of communication between MDTs and parents. Our methodology and results are potentially transferrable to the management of other long-term conditions. More research is needed to define the complex intervention of shared caring in CKD management, and to begin the process of developing and testing a multidisciplinary intervention to help professionals and parents collaboratively identify parents’ support needs and preferences for shared caring.

### Limitations

Because of the small sample size and the condition-specific focus of the study, the results are a ‘snapshot’ of one clinical situation so while we propose that our results could potentially be applied to other clinical specialities, we make no claims to our results being generalisable to other settings. This study focuses on parents but we recognise that they may share clinical care with their children who have CKD and that some children may help parents with aspects of treatment, or translate for them if parents’ first language is not English. However it was beyond the scope of this study to focus on children’s contributions to their own clinical care.

## Endnotes

^a^Identification of a small number of individuals with required characteristics who are then used as informants to identify others for inclusion in the study. In turn, these informants are used to identify further participants. An example of non-probability sampling.

^b^Step down to the patient hotel, an MDT, staged-approach designed to help parents learn to safely deliver a clinical task, such as set up dialysis in a new environment with minimal support from experienced staff and in preparation for taking the child home and ‘going solo’ with clinical care

## Abbreviations

CKD: Chronic-kidney-disease; MDT: Multidisciplinary team; PI: Principal Investigator; REC: Research Ethics Committee.

## Competing interests

The authors declare that they have no competing interests.

## Authors’ contributions

VS led study conceptualisation, design, the funding application, data collection, analysis and drafted the manuscript; DA and JW contributed to conceptualisation, design, the funding application and data analysis; DA, JW, HL, NJAW,TS,LW and LQ contributed to design and analysis; RN undertook fieldwork and data analysis in Phase 3, all authors read, contributed to and approved the final manuscript.

## Authors’ information

The authors include researchers and clinicians and represent many of the health disciplines involved in renal MDTs. VS, TS and DA are nurses; HL and NAJW are doctors, LQ is a dietician; LW is a Clinical-Psychologist. VS, DA, JW and RN are experienced qualitative researchers, VS is an experienced mixed-methods researcher with expertise in using Framework Analysis, and DA and JW are experienced ethnographic researchers. JW leads the Social Theory of Learning group at the University of Manchester.

## Pre-publication history

The pre-publication history for this paper can be accessed here:

http://www.biomedcentral.com/1472-6963/13/264/prepub

## Supplementary Material

Additional file 1 Supplementary data from Phase 1. Click here for file

Additional file 2 Examples of tools used by professionals when teaching parents. Click here for file
