# Supplementary material for: Multidisciplinary teams, and parents, negotiating common ground in shared-care of children with long-term conditions: A mixed methods study
Source: BMC Health Serv Res. 2013 Jul 8;13:264. doi: 10.1186/1472-6963-13-264 (PMC3720539; doi:10.1186/1472-6963-13-264)
Supplement: Additional file 1 — Supplementary data from Phase 1. [file 1472-6963-13-264-S1.docx]

Additional File1: Supplementary data Phase I: *Engaging parents in shared practice*

| ***Discipline** | **Establishing parents’ condition-related knowledge and position on ‘renal journey’** | **Determining parents’ learning potential** | **Prioritising skills/knowledge to teach** | **Defining/meeting individual learning need** |
| --- | --- | --- | --- | --- |
| CP | Telling them I… having an illness is hard work, difficult & quite challenging so I’m here to add a bit extra if they’d like it - as part of the team, let them know I‘d be involved with most families at some point .. normalise it |  | I have a model which is the child in the middle, the family, the team and the wider culture…we have life going on here and we’ve got history here and impact of disease here… | Might say to them ‘Well how are you finding out things…are we looking after you ok..you know, are you someone who likes to know everything in a big heap?’ |
| Dr | You realise drip feeding [information] is all you can do because the environment isn’t great. need formal times, where you can create a better environment, on specific things which have objectives and backed up by informal meeting, You often see [colleagues] walk past [parents] on the ward when they’re going to see someone else and there’s been a sort of, reaction there [from the parents] where they’ve [colleague] been able to reinforce things | If a child’s unwell you are not always able to have an interpreter present when you need one and that can be a problem  Probably our biggest challenge is getting them [parents/children] to understand and do things, particularly in chronic conditions they’re going to live with for 50 years, they really do need to know | Where there are problems with comprehension or communication, then what you do is strip out things you think are essential to the child's immediate care and try and get those done first… think later about other things, e.g. we have patients on dialysis who the nightmare might be teaching them dialysis, and you think ‘ teaching them to take BP will wait’, even though that's a basic part of the whole process | It might just be one thing which I think is important to make the point that "it’s not quite that, it’s actually this" and then leave everything else which wasn’t quite correct…especially if it’s a chronic patient who going to be ?with us for a long time there’s going to be plenty of opportunity … |
| Dtn | ..easy to fall into trap of assuming people understand things..they want to give good impression of following everything but [maybe] haven’t quite grasped things. Got to create a bit of a rapport with them | Assess how much information they are taking in. As part of the assessment] I’ve had a meeting with two school nurses, a community nurse, cook and a class teacher |  | …attempt to slow things down and make things as simple as possible |
| PW | …building a relationship, you’re not going to get anywhere until that’s happened | …might use dolls, art and craft, or just talk, drawing, whatever way of learning going to be best for them |  | have an agreement with parents, ‘ is it ok if we go to another area to discuss things [with child]?’ and most are more than happy with that. Some parents might want to know what you’re going to say..and we can let them know, we can discuss this … |
| Ph | …sometimes people come in with preconceived ideas like their auntie’s on steroids for something, or granny takes aspirin | When I’m going over medicines I’m like ‘you’re not taking in a word I’m saying,’ I can understand it because they don’t have to go out that room and use that information, but the whole point about it is for them to even have it explained initially, because what I tend to do is go over it once, then again, then I say I won’t speak to you again until discharge, then it will mean something to you for real, because you’ll be used to them on the ward | I think it depends on how long you’ve got with the parent … | …in the pharmacy where you’ve maybe tagged on to the end of something else to explain about drugs and presume that they understand because it’s what we’ve told everybody else and they’ve kind of got on with it. |
| SW | I’d do home visits and then you get quite a different or perhaps more accurate picture of what people are like…it’s good for them because they don’t feel like they’re burdening us (SW) too much ……give people confidence to feel it's ok to ask whatever questions they want to ask and to also not to know necessarily. | There’s a lot of texting [with parents] and actually that’s quite good because..organise Disability Living Allowance for families. It’s actually a very good way of getting to know families… take a little time to find out whether people don’t seem to be responding because they might be in shock or .. | Because there might be some sort of learning difficulty, so that’s not easy to, sort of, establish first of all, it can take a little bit of time. | We’re led by them, if we can we don’t rush things It’s even more sometimes than just knowledge, they might not feel that they’re able to take on tasks that we feel would be in the child’s best interests, e.g. dialysis, some parents are really resistant to taking what they see as medical treatments into their own home |
| T | …actually trying to experiment with the parents with different ways of telling their story that will get people to hear it, because they are trying to find their way through the system | .. they know the information they have been given but actually they really feel it is so unfair and it shouldn’t be true…sometimes just saying that [to parents] is a relief because it gets them a bit acknowledging how bloody awful it is and that is it and they have to deal with it, | You can see a parents’ behaviour affecting the child’s behaviour and then you can adjust the parent’s, if they are being too pressuring to get their child to do something and if you get them to back off a bit and give a bit more responsibility to the child, |  |

*Key for Additional File 1: CP=Clinical Psychologist; Dr=Consultant Paediatric Nephrologist; Dtn=Dietician; N= Nurse Consultant, Advanced Nurse Practitioner, Clinical Nurse Specialist, Sister, Staff Nurse; PW=Play Worker or Play Specialist; Ph=Pharmacist; SW=Social worker; T=Family Therapist or Therapist.

Supplementary data Phase I - *Knowledge exchange and role negotiation*

| **Discipline** | **Teaching in stages** | **Using metaphors/tools** | **Assessing understanding** | **Reviewing learning progress** |
| --- | --- | --- | --- | --- |
| CP | We talk to parents about letting the child start to organise their own medicines with supervision, telling them how we expect their role to develop. We do get some resistance, they've been in control for a long time, it's very hard to let go of the reins |  | …one of the ways of [assessing parents’ understanding] is one of us or various people in the team will go and ask the parents, "How do you take on information? How would you like us to give it to you? | OK so what did they [Dr] say, what was it like for you, how could it have been different? |
| Dr | Looking at the complexity of it [information] and whether it can all be done [taught] in one go or if it needs to be given in ‘chapters’  We tend to do everything in little bite sized chunks really | ...Nutritional Care did a series of photographs and showed them, and then had to show them pictures of what the numbers looked like because they said, “You can’t count but the numbers should look like that. If you keep pressing until you get one that looks like that”. So they have a sort of a like a strip of pictures. And they were just told, “This is what it’s got to look like”. | …don’t think we take any formal assessments of families …l start talking about whatever we need to, start with …we pick up the clues and verbal feedback that we’re … it’s not until they’re seen again when they have an opportunity to see another member of the team that you realise how much they’re taking in .. frequently, you meet with both parents and that does help me to pitch…, | I say, well you might see somebody else, you’ll go and see Dr A and you may suddenly think, aha, now I know what Dr B was talking about! |
| Dtn | We’ll just go through the major hits…’okay, this, this and this, these are the things you have to avoid tonight and tomorrow, then we’ll send the rest of the information by post | …you talk about what they’re eating and any specific changes they have to make and then I give them the leaflet so they can read it when they get home. |  | …because doctors are always asking, do they understand what the medications are for all the time I guess, we do that informally all the time as part of our routine reviews that we do with them. |
| N | When we do our teaching ‘sessions’, we try and stage them over a couple of weeks so people have time for one thing to go in before you layer on another ,say right where did we get to last time? This is what we’re going to be saying this time, before you say it again and at the end | …so when she goes to the local hospital now she can give that [an ‘All about me book’ ]to them and say right, read this, I’m going to go away for ten minutes and come back and then we can start again. To give parents the information we use some tools we give to the children | …important whenever you start something you go back and check, say a family had a diagnosis or treatment in clinic, quite big news and it’s going to have an impact in their family life, they are going to take on care at home, where possible I’d try and go and see them at home with the SW.. pick out what was mentioned in clinic, because you find people hear one bit of news and that’s it... | It’s trial and error really… it’s not something I consider I’ve been taught how to teach [parents]. Not only the ability for people to take on information, some parents do have some learning needs of their own and perhaps, as {colleague in focus group] was saying, perhaps they don’t understand all the jargon and the complexities of some things, so things need to be simplified from a cognitive perspective as well. But it [ talk back] works very well with the parents |
| PW | had a parent saying their child has never had a blood test, didn’t know what to expect, so I sat down with the child [and parents] and just went step by step.  In the ward round it’ll be planned when they’re going home, who’s going to go through things with them, | I've got my book of pictures and my dolls…parents get quite interested in them. but sometimes you take then & you're just talking to the adults…when doing transplant perhaps, about what's going to happen… everything needs to be simplified .. you think you're doing that, but we're so used to living in the hospital world.. |  | Once you’ve taught the parent who couldn’t read and he had a good memory so we just made sure in clinic that we always stressed, verbally, you know, and he would repeat back to us what he needed to do and also we can demonstrate on syringes how much medication we’ve got to give and things like that. |
| Ph | Depends on how long you’ve got with the parent, if you’ve got a set amount of time and you’ve got some info to get over to them | So you feel you’re actually giving them some form of information [renal medicines book] , and a lot of times they get to know them very well therefore don’t need to use the book as much, but at the beginning we find it very helpful, |  |  |
| SW | …check where they feel they're at, gauging all the time where we've got them to, where we want to get them to, we might want to bring them to a level of knowledge they’re not ready for, if not ready to do injections, gastric tube you might get a comm.. nurse to do it, try to find solutions rather than force them to take on tasks they're not ready to learn |  | One of the key things … maybe for parents that have been in the system a bit longer, is the need to recognise how expert a parent often is in their child's own care. And therefore to kind of hear the parent's perspective and where they're up to rather than just telling them what they should be doing next. |  |
| T | Often try to move the conversation to somewhere where the parents maybe feel more competent |  |  |  |

Supplementary data Phase I - *Promoting common ground*.

|  | **Role allocation/differentiation** | **Interpretation** | **Acting as brokers** | **Sharing information within the MDT** |
| --- | --- | --- | --- | --- |
| CP | when I first meet people will say ‘So what’s going on for you…that gets them saying what they’ve taken in, because I’ll know probably what they’ve been told and a lot of what they need to know..will also give me an understanding of ok they haven’t taken that bit on board or haven’t understood…I’ll go back to the team who will know and say ‘this is what they think is happening, is that right? |  |  | Or I might not know what they've understood but I'll go back to the rest of the team who will know and say, "This is what they think is happening, is that right?" |
| Dr | for some things we have a check list, like, when they’re going on to dialysis, that they will have to go through but, you’ll be going backwards and forwards on that check list and going through things over and over. Some things are still a bit of ad hoc, but there is a list that we have in our minds that we want to make sure that parents are competent in all aspects and that actually…not just the medical condition that they’re supported for but also the psychological and the whole patient | The tariff that you get for episodes of health care, you don’t get a different amount of money if you’re dealing with a family where they don’t speak English. So it’s…yeah, it can be quite difficult . Family members as interpreters... good and bad... some really positive things in it... but... sometimes they get very embroiled in the whole thing and don’t have that degree of objectivity that you’d like to have in an interpreter... They’re often trying to protect or to help them… | We provide a big structure of psycho social support..it really is a presence of a team. She [nurse] might get help from [name], her colleague, and she might get help from other people on the ward, other nurses on the ward, or from the pharmacist or a dietician, you know, they certainly do it as a team. The dialysis, this is a lot less about parents themselves providing the training, it tends to be, you know, more an explanation by the doctors and then, the nurses | We are very like minded in that respect. not having a full compliment in a MDT that’s had a huge impact on how that team works and it does have an impact on the patients as well. So actually having the full complement makes a huge difference, it just functions much better and parents find it easier having everyone to contact. So the great thing about our team, I think, is, that we do all talk to each other, there’s not this hierarchy and we all value each other’s opinions and we do listen to each other. |
| Dtn | worked as a team, it took a long time, because when you set the boundaries, you know, we back them up and we got there eventually, some things are still a work in progress but… from my perspective parents may lose out on the ad hoc conversations. But I have a more in depth conversation when I do see them, so I think it may even out over time. | We need interpreters, we’ve got quite a lot of multi-cultural families  ...lose the casual corridor chat | I hear what the Drs are saying and the Drs hear what I’m saying  We’re a close knit team | We worked at as a team, we got there, it took a long time, you know, but it was, you know, it was setting the boundaries because when you set the boundaries, you know, we back them up and we got there eventually, some things are still a work in progress. |
| N | nurses have a very definite role to teach people how to do things, the higher end scale who are going to have a lot more clinical input we would definitely go in as two people, because I don’t think as a nurse you have necessarily got the skills to think about [all the MDT members’ contributions], we will encourage them to meet all the team as they go through, so everyone will get offered if they want a psychologist, it might be a one off visit with the psychologist, but we will introduce them to the social worker, | …not always best to use the family [to interpret] as they might not understand what you’re saying anyhow…do actually get interpreters in,… so it’s easier for us to pick up the teaching they need ,or they may have a piece of information that you’re not aware of before you start teaching so, yeah, that’s a real benefit.  We have in house interpreters not only will they interpret exactly what you’ve said, but they’ll tell you everything that the parents have said, | Sometimes [if a psychologist is mentioned]. people are wary maybe that they are going to tap into emotions that they don’t want to express. Social workers less so…sometimes you have to explain the role of SW here. People have a vision of them from child protection but we have explained the supporting role. I think sometimes the SW is on the back of support, disability, around financial support, our social worker sorts that out, builds up very good therapeutic relationship with a lot of families. | Consultants are very good about not being pressurised into discharging someone sooner than what we would want really. We would never send someone home on a Friday on dialysis for first time  Some teenage mums… have done fantastic, much better than some of the, you know, two point two, , children and parent families that you think are going to get to grips with it and just fall apart whereas somebody, you know, the teenage mums have coped amazingly so … each of us will pick up something different from the same consultation |
| PW | Its more about what we [play workers] can do separately, have got community play specialists, I go into houses with the nurses and work with children …while the parents get to relax while not talking in front of their children about things they may find upsetting, the methods I use for the children sometimes are very helpful for parents - are visual and simple |  | I go into schools to explain what the child has had done…you know, the catering staff and say this is the sort of diet this child needs.  …you know, schools can plan it. The dietician can be helpful to get in touch with the school. I sat down with the child and went though it [blood sampling] … |  |
| Ph | So it’s [post clinic meeting] a really good place to share information and to make sure that we are all saying the same thing as well | We have some information in various languages especially with regards to transplantation and organ donation, it’s very key to make sure you’re hitting all nationalities there, but the main thing is the communication and I think it’s important that families are offered the same level of information and you have to use a variety of other tools for non English speaking families |  |  |
| SW | if you can find out whether they’re finding it difficult to take on board the information or whether it’s the…they’re listening to information and thinking, I need to get back and pick the kids up from school or there’s other things going on, it makes them present as if they’re not taking it in but actually it’s finding out what else is going on in | You can’t just wander in and chat to them [if an interpreter is needed] [mother] can’t read, not even the basics. But, I mean obviously she’s got her own way of dealing with that…We don't have enough interpreters. | We can liaise with the [parents’] employers, with large kidney charities to help out with things, you know hospital stays…transport in and out of hospital… |  |
