# Supplementary material for: Multidisciplinary teams, and parents, negotiating common ground in shared-care of children with long-term conditions: A mixed methods study
Source: BMC Health Serv Res. 2013 Jul 8;13:264. doi: 10.1186/1472-6963-13-264 (PMC3720539; doi:10.1186/1472-6963-13-264)
Supplement: Additional file 2 — Examples of tools used by professionals when teaching parents. [file 1472-6963-13-264-S2.docx]

Additional File 2: Examples of tools used by professionals when teaching parents

| **Type of resource** | **Examples** |
| --- | --- |
| Written information | - Medication sheet - Inpatient/ward drug charts - Leaflets about conditions - Step by step guidelines to setting up dialysis machine (parents encouraged by professionals to highlight text, add notes to make the guidelines personal to parents’ learning needs) - Home dialysis recording chart - Information sheet with contact numbers e.g. ward - Booklet about dialysis machine - Recipe for feed - Pre- and post-transplant information |
| Visual information | - Photograph books showing children having procedures and treatment - Child growth chart - Posters e.g. exit site, diffusion through a tea bag - Spontaneous drawings e.g. peritoneum; fibrin in dialysis fluid; how dialysis fluid works; graphs to demonstrate effects of home dialysis vs. in centre dialysis; catheter tube - Diagrams about hand washing - Computer graphs showing child’s blood results e.g. creatinine levels |
| Equipment | - Syringes - Medication - NG tube - Teddy bear with catheter - Catheter for dialysis - Dialysis machine - Dummy of peritoneum with exit site and catheter - Packs of dressings - ‘Activity pouch’ (to keep exit site dry) - Bags of dialysis fluid |
| Meeting others | - Visit to ward - Other child having same treatment e.g. home dialysis - Other parents whose child is having the same treatment e.g. home dialysis |
| Self help | - Self help Internet - Talking with family, friends and neighbours - Reading books/information etc |
| Metaphors and comparisons | - **Parents learning home dialysis:**   Nurse asks parents: ‘Why would no fluid come out?’  Mother: ‘He’s dehydrated, he’s had a wee’  Father: ‘The clamp is on. The position he’s lying in’.  Nurse: ‘Yes, it’s like drinking juice from a carton, when you get to the bottom you have to shake it to get it out. We’ll talk about him needing to change position’   - **Doctor talking to parents about their child’s fluid requirements:**   Doctor reassures: ‘More water is fine. If he wants it give it to him. When he is ill, his sodium will go up and he’ll want to drink more. It’s like when you eat a packet of crisps in the pub, you want to drink more. That’s why they have crisps, peanuts, things like that. When he’s unwell, he will produce more weak urine, and so his blood concentration will go up, his salt will go up and he’ll want to drink. When he’s older, he’ll be able to tell you ‘mum, I’m thirsty’, but he can’t now’   - **Nurse talks in an interview about teaching parents’ about dialysis bags:**   ‘When I talk about dialysis bags and the different concentrations and when you talk about the stronger dialysis bag basically pours more water out of the body, so when you’ve got very dilute blood and you’ve got a concentrated solution the body likes to be in an equal balance the water gets pulled across to dilute that sugar down till it’s at an equal concentration either side of the membrane… and when I talk about it with families, depending on their capacity or whether they’re looking a bit confused, I can talk about normal squash and the double concentrate that’s now available and if you put the double concentrate to the peritoneum it’s like the strong bag, you would need double the amount of volume of water to be pulled across to make it drinkable’   - **Parent asking nurse about infections resulting from dialysis:**   Mother: ‘How do you know if she has peritonitis?’  Nurse: ‘By the colour of the fluid. It’s usually very clear, but if there’s an infection, you might get bits in it, like if you shredded a cotton wool ball and put it in water, it’s called fibrin. That’s protein from the peritoneum’ |
